# Vascularization is the next challenge for skin tissue engineering as a solution for burn management

**DOI:** 10.1093/burnst/tkaa022

**Published:** 2020-08-03

**Authors:** Hady Shahin, Moustafa Elmasry, Ingrid Steinvall, Folke Söberg, Ahmed El-Serafi

**Affiliations:** 1 Department of Hand Surgery and Plastic Surgery and Burns, Linköping University Hospital, 581 85, Linköping, Östergötland, Sweden; 2 The Department of Biomedical and Clinical Sciences, Linköping University, Linköping University Hospital, 581 83, Linköping, Östergötland, Sweden; 3 Faculty of Biotechnology, MSA University, 26 July Mehwar Road, 125 85, 6th October City. Egypt

**Keywords:** Burn, Skin, Regeneration, Tissue engineering, Vascularization

## Abstract

Skin regeneration represents a promising line of management for patients with skin loss, including burn victims. The current approach of spraying single cells over the defective areas results in variable success rates in different centers. The modern approach is to synthesize a multilayer skin construct that is based on autologous stem cells. One of the main complications with different types of transplants is sloughing due to the absence of proper vascularization. Ensuring proper vascularization will be crucial for the integration of skin constructs with the surrounding tissues. Combination of the right cells with scaffolds of proper physico-chemical properties, vascularization can be markedly enhanced. The material effect, pore size and adsorption of certain proteins, as well as the application of appropriate growth factors, such as vascular endothelial growth factors, can have an additive effect. A selection of the most effective protocols is discussed in this review.

## Background

Skin is the largest organ of the body and plays a vital role in homeostasis. The loss of merely 15% of the total body surface area (TBSA) is sufficient to be considered as life-threatening. Impairment of the skin’s barrier function, as a consequence of mechanical, chemical or thermal injury, can induce substantial water loss that may lead to hypovolemia [1, 2]. Massive skin burns induce severe disruption of the body’s homeostasis, including hypermetabolism, immunodepression and vascular hyperpermeability, which promotes edema formation [1].

Advancements in resuscitation techniques, infection control, nutritional support and surgical care allowed the survival of patients with burns to >90% of their TBSA [1]. The survival rate became even higher in children, with up to 50% enhanced survivability with 95% burns of TBSA [3]. The enhanced survival was associated with the emergence of novel solutions for wound coverage that tackle later complications, limit fluid loss, control pain and reduce the risk of hypertrophic scarring [4].

Early intervention has a considerable impact on wound recovery, restoration of functionality and aesthetic outcome [5]. On the other hand, delayed wound re-epithelialization would not only increase the risk of infection but can also be detrimental to the functional outcome and skin appearance. Delayed re-epithelization can lead to contractures, hypertrophic scarring, psychological stress, social isolation and longer hospital stay [6]. Therefore, the establishment of a management line that can enhance the early wound re-epithelialization is an attractive target for burn care management.

The first line of intervention in burn management is tissue debridement, which involves excision of the damaged skin as well as the affected surrounding tissues. Debridement facilitates wound healing by removing necrotic tissue and contaminants, which decreases bacterial load, suppresses the inflammatory reaction and creates an adequate bed for eventual grafting or biologic dressing [7]. However, the excision can create raw areas of open wounds, which gives rise to the challenge of essential quick covering.

Over the years, various strategies have been established that ultimately attempt to regenerate the barrier function of the epidermis by covering deep and extensive burns while sustaining the aesthetic aspect to an acceptable degree [1]. The golden standard is to harvest split-thickness skin biopsies from unburned areas and graft them onto deep burn wounds. This technique involves the creation of another wound at the donor site, which resembles superficial second-degree burns. The donor-site wound can heal in 2 weeks without apparent scarring. While autologous split-thickness skin grafts are likely to be the treatment of choice in large second-degree and third-degree burns, the lack of suitable donor tissue in the most severely injured patients emphasizes the demand for alternative temporary or permanent wound coverings [4]. Furthermore, regrafting requires relatively long waiting times for the donor sites and grafted wounds to heal before harvesting additional split-thickness skin [1].

Skin tissue engineering holds promise as a rapidly expanding field in reconstructive surgery as well as burn management. The concept emerged in the 1980s, with the employment of confluent keratinocyte layers to achieve full restoration of the epidermal barrier [8, 9]. Subsequently, dermal substitutes were introduced to target the missing dermis in full-thickness wounds [10]. At the present time, such developed dermal alternatives are widely used for the treatment of full-thickness burn wounds with promising outcomes in terms of pain relief, healing and aesthetics. The modern approach combines an epidermal and dermal layer by integrating keratinocytes and fibroblasts into an acellular matrix [11–13]. The limited sizes of tissue constructs grown *in vitro*, as well as the subsequent challenge of integrating these constructs *in vivo*, remain major obstacles in the field of tissue engineering. Naturally, most cells would be located within the proximity of no more than 100–200 μm from the nearest capillary, with this spacing providing adequate blood supply, which, in turn, enables diffusion of oxygen, nutrients and waste products to sustain viable tissue [14]. Similarly, when tissues grown in the laboratory are implanted into the body, limited diffusion resulting from inadequate vascularization allows only cells within the same proximity from the nearest capillary to survive [15]. Establishing an adequate and sustained blood supply is crucial for accomplishing functionality of skin constructs *in vivo*. Upon success, the routine coverage of extensive full-thickness skin wounds with functional synthetic skin constructs may become applicable in clinical practice [16].

## Review

### The concept and challenges of cell-based therapy in burns

Over the last two decades, major progress has been made in the field of skin tissue engineering, inspired by the typical insufficiency of skin grafts. Autologous cell spraying emerges as the first regenerative solution for such a dilemma. This procedure involves the use of a suspension of cells isolated from a patient’s skin biopsy. The epidermal cells are enzymatically extracted and suspended in an aerosol-based solution that is sprayed over the skin bare area [6, 17, 18]. Although this method showed to accelerate the re-epithelialization, the results are controversial and lack standardization and reproducibility [19, 20]. In the areas where dermis is lost, the newly formed epidermal layer lacks support as it can easily break down under pressure. As a consequence, cells and matrices were integrated in a number of different strategies to introduce bioengineered skin, tissue-engineered skin constructs, bio-synthetic skin substitutes, bio-constructs and other terms [21, 4]. These solutions aim to restore the intact barrier that protects the wound from infections, provides pain relief, enables temperature control and prevents fluid loss from the wound surface. An ideal tissue-engineered skin construct must fulfil three major requirements: safety for the patient, clinical effectiveness and convenience in handling and application. In addition, such bio-constructs must be non-immunogenic, non-toxic and not predispose to the transmission of infectious diseases. The implicated biomaterials should be biodegradable, repairable, easy to manufacture and offer similar physical and mechanical properties to the skin [22].

The list of challenges to the successful uptake of skin constructs includes poor mechanical integrity, immune rejection and failure to integrate with the supportive layers; the major challenge is inadequate or inconsistent vascularization [22, 9, 4]. Although acute hypoxia typically induces the healing responses of fibroblasts and keratinocytes [23, 24], persistent hypoxia decreases keratinocyte migration and proliferation, leading to fibroblast dysfunction and eventually tissue loss [24–26]. When combining several layers in a skin construct, the cells away from the wound surface can suffer from hypoxia. Hence, inadequate vascularization is a major hurdle that hinders the clinical use of dermal constructs as it leads to partial necrosis, loosening or infection of the substitute [27].

During early gestation, the vascular tree starts to develop when angiogenic cells form clusters that merge into solid tubes. These tubes canalize to form blood vessels that are covered with angioblasts as the outer layer of their walls [28]. After this, vasculogenesis occurs, which involves the imminent maturation of these precursor angioblasts into endothelial cells (ECs), forming a *de novo* vascular network. These minute, capillary-like vessels eventually differentiate into either veins or arteries [29]. The natural development of the microvascular tree involves repeatedly dividing arteries into smaller vessels, known as meta-arterioles (80–100 mm), which leads to the formation of capillaries (10–15 mm). These micro-vessels tend to further divide into numerous smaller branches, thus maximizing the available area for nutrient exchange [30]. The capillaries tend to fuse together forming post-capillary venules, venules and, finally, veins.

### Factors affecting vascularization of tissue-engineered skin constructs

Efficient vascularization strategies are vital for skin implants to achieve their biological functions and a major prerequisite for the safe application of tissue-engineered skin in clinical practice [16]. Failure to provide an adequate blood supply could result in total/partial necrosis, which might lead to infection, sloughing of the implant and sepsis. As a result, much attention has been devoted to the stimulation of vascularization in engineered skin implants [31]. The strategies for vasculogenesis could be classified into pre-vascularization and angiogenic approaches. The latter is based on promoting the ingrowth of blood vessels in implanted skin substitute. However, due to the delayed growth of newly developing micro-vessels, with a rate of approximately 5 μm/h, they have proven unfit for vascularizing large implants [32]. The pre-vascularization approaches involve generating micro-vessels within tissue beds prior to grafting, resulting in a more instantaneous blood supply [33]. Multiple factors can contribute to the vascularization of skin constructs, which are summarized below and in Table 1.

**Table 1:** Summary of the factors that can contribute to vascularization of skin constructs

Example	Attributes	Reference
*Scaffold-based vascularization*		
Artificial skin replacement films (e.g. Matriderm® & Integra®)	Mimic the natural dermal layer Provide highly dense micro-vascular networks Produce better structured neodermis Stimulate an angiogenic tissue response Clinical effectiveness is diminished in time Delayed vascularization Increased risk of infection	[34–37, 100, 101]
Fibrin and/or hyaluronic acid scaffolds	Did not accelerate wound closure	[40]
Surface activation using argon/plasma	Enhanced angiogenesis Accelerated neovascularization	[37, 38]
*Proangiogenic growth factors*		
Hydrogels with plasmin-degradable VEGF-secreting nanocapsules	Accelerated wound healing Enhanced vessel maturation Decreased fibrotic response	[40]
Hydrogels with basic fibroblast growth factor-loaded alginate microspheres incorporated into carboxymethyl Chitosan-poly vinyl alcohol-based scaffold	*In vitro* Higher bFGF bioactivitiy Promoted cell proliferation *In vivo* Accelerated wound healing Faster re-epithelialization and regeneration of the dermis Promoting neovascularization Higher density of mature blood vessel *in vivo*	[41]
*Gene-activated matrices*		
VEGF plasmid DNA incorporated into a collagen-chitosan membrane scaffold	Increased density of newly formed and mature blood vessels Improved regeneration of the dermis The tensile strength of the repaired skin reached up to about 80% of the normal skin	[43, 44]
Polyplexes basic fibroblast growth factor-encoding plasmid (pbFGF)-loaded fibrous mats	Sustained release of pbFGF for 4 weeks Higher wound recovery rate in diabetic rats Improved vascularization Enhanced collagen deposition and maturation Complete re-epithelialization and formation of appendages	[45]
Gene-activated dermal scaffolds using VEGF gene vector protected by a copolymer	Increased vascularization of full-thickness skin wounds in nude mice Unstable vessel formation in the scaffold	[47]
Bioactive scaffold-like membrane based on biodegradable Poly*-N-acetyl-*glucosamine nanofibers	Enhanced wound healing Enhanced VEGF-driven angiogenesis and blood vessels formation in the newly synthesized tissue	[48]
*Angiogenic growth factor inducers*		
Copper-doped borate bioactive glass microfibers	Induced HUVECs migration and proliferation *in vitro* Promoted the formation of elongated tube-like structures Stimulated VEGF secretion of the fibroblasts Accelerated the healing of full-thickness skin wounds Increased number of mature vessels in wound beds at day 14 post-surgery Improved deposition and arrangement of collagen fibers in a fashion resembling normal skin No account on the long-term effect of copper *in vivo*	[49]
Poly L-lactide-co-glycolide mesh integrated with collagen-chitosan scaffold	Promoted tissue regeneration and vasculogenesis when combined with split-thickness autografts in treating full-thickness skin defects in rats	[50]
	Enhanced the tensile strength of the repaired skin reaching up to 73% of normal skin 8 weeks after implantation Promoted angiogenesis and increased the density of micro-vascular networks the newly formed skin	
Customized dextran-based hydrogel scaffold	Promoted angiogenic response when tested in treating full-thickness burns in mouse models Epithelial repair occurred with hair follicles and sebaceous glands	[51]
*Cell-mediated angiogenesis*		
Human dermal microvascular endothelial cells with fetal fibroblasts in 3D constructs	Faster healing of second and third degree burns in young children without auto-grafting Aesthetically satisfactory results	[53]
HUVECs on an artificial dermis	Enhanced microcapillary formation and organization of endothelial cells	[58]
HUVECs co-cultured with keratinocytes and/or fibroblasts on collagen with or without glycosaminoglycan	Spontaneous formation of capillary-like tubes *in vitro.* Enriched vascular density of the tubular structures connecting between the capillary-like meshwork	[59–61]
Local or systemic administration of endothelial progenitor cells	Integration of the cells in the capillary wall at the wound or ischemia site The new vessels exhibited contractile and vasomotor activity	[64, 66, 67, 72]
Human blood outgrowth endothelial cell integration in a dermal construct	Adequate vascularization and re-oxygenation of the wound bed with better epithelization and matrix organization	[25]
Human microvascular endothelial cells-seeded fibrin-based micro-tissues	Robust endothelial sprouting of larger vessels which extended for a relatively long distance (1–2 mm)	[62]
Bone marrow mesenchymal stem cells-conditioned media	Promoted *in vitro* proliferation and migration of endothelial cells Local injection *in vivo* enhanced collateral perfusion and improved limb function	[82]
Bone marrow-derived mesenchymal stem cells in scaffolds	Regeneration of dermal, fibrous, fat, and vascular tissues in animal models Accelerated burn and wound healing in patients	[81, 83, 10]
*In vivo* administration of human adipose-derived mesenchymal stem cells	Increased microvascular density	[87]
Human umbilical cord blood-derived mesenchymal stem cells	UCB-MSC enhanced the regenerative capacity of skeletal muscles when engrafted in an ischemic hind limb mouse model The role in angiogenesis is controversial	[89, 91, 92]

*VEGF* vascular endothelial growth factor, *pbFGF* polyplexes basic fibroblast growth factor, *HUVECs* human umbilical vein endothelial cells, *UCB-MSCs* human umbilical cord blood-derived mesenchymal stem cells

#### Physical properties of dermal scaffolds

Scaffold-based vascularization strategies have been studied extensively in the field of skin tissue engineering. Dermal scaffolds have the capacity to mimic the natural dermal layer, which can provide stability as well as highly dense microvascular networks that nurture the overlying layer of keratinocytes [9]. The implantation of artificial dermal scaffolds typically stimulates an angiogenic tissue response, involving the ingrowth of newly formed micro-vessels. Furthermore, Schneider *et al*. demonstrated that angiogenesis can be stimulated with implanted artificial skin replacement films, such as Matriderm® (Dr. Suwelack Skin & Health Care AG, Germany) and Integra® (Integra Life Sciences, USA) when introduced into full-thickness skin wounds in rats [34].

Furthermore, structural adjustment of scaffolds by altering their pore sizes and interconnectivity may improve their angiogenic tissue response [16]. Choi *et al*. demonstrated that scaffolds with pores size <200 μm were found to enhance generating vascular networks with smaller vessels at higher density and superficial penetration. Unfortunately, most of these capillaries are blocked by the polymer backbone. On the other hand, pores with sizes >200 μm were associated with larger blood vessels and deeper penetration, which is preferred for developing large 3D tissue constructs. The configuration of the vessel is more physiological with the larger pores, as they allow the migration of other cells necessary for the proper development of the capillaries, such as fibroblasts [35].

It is not only these structural aspects, but also the physico-chemical properties of the scaffold material that can have a profound influence on the vascularization of scaffolds. Pre-treatment of the implant surface by plasma was suggested as a promising activation technique to enhance the angiogenic tissue response to various implants. Matriderm® is a bio-scaffold, consisting of bovine ligaments and dermis with pore sizes of 20–150 μm and non-cross-linked elastin hydrolysate [36]. Ring *et al*. attempted surface activation of Matriderm® with low-pressure argon/hydrogen plasma and applied it in mouse dorsal skinfold chambers. Plasma can change the surface polarity, which enhances protein adsorption and, consequently, cell adhesion. Faster vascularization and improved angiogenesis were observed with plasma treatment in comparison to non-treated controls [37]. Subsequently, more efforts have been exerted in developing novel dermal scaffold prototypes with enhanced intrinsic bioactivity rather than merely modifying commercially available ones. Polyurethane nanocomposite scaffolds were exposed to argon plasma for 5 minutes and combined with adipose-derived stem cells. This combination was associated with better vascularization and tissue integration with the dorsal subcutaneous tissue of the rats, in comparison to scaffold without plasma or without cells [38]. The preparation of the scaffold with plasma is not straightforward, however, as it needs a specific bioreactor and radiation safety procedures [37, 38].

#### Angiogenic growth factor

##### Direct incorporation of proangiogenic growth factors

The incorporation of angiogenic growth factors, including platelet-derived growth factor (PDGF), vascular endothelial growth factor (VEGF) or basic fibroblast growth factor (bFGF) is considered an attractive strategy to enhance the ingrowth of blood vessels into scaffolds. However, free and random infusion of these growth factors to the wound inevitably lead to rapid loss of their bioactivity because of impaired protein stability at 37°C. Therefore, sustained release of these factors into the wound is preferred [39]. Cam et al. demonstrated that bioengineered VEGF-secreting hydrogel nanocapsules, composed of hyaluronic acid or fibrin, enhanced vessel maturation and wound healing shortly after implantation in comparison to VEGF-free hydrogel [40]. Several strategies have been investigated to achieve sustained release of growth factors from scaffolds, where biodegradable microspheres offered the most accurate release control. In skin tissue engineering, gelatin, poly L-lactide-co-glycolide (PLGA) and alginate-based microspheres proved to significantly ameliorate the neoangiogenic response of the host tissue to dermal scaffolds when loaded with the required growth factors [41].

##### Incorporation of proangiogenic growth factor gene

The concept of a gene-activated matrix involves the pre-treatment of matrices with gene vectors to achieve a sustained DNA delivery to the ingrowing cells at the implantation site [42]. This approach improved skin regeneration and vascularization in porcine and rodent models when activated dermal scaffolds were supplemented with plasmids encoding for VEGF [43, 44] and bFGF [45] were used. In another interesting approach, a collagenous matrix was used for long-term application of a viral vector for gene therapy in clinical trials. An adenovirus encoding for PDGF was applied to the matrix following debridement of diabetic ulcers. This method was showed to be safe and effective in a phase I/II clinical trial [46]. Nevertheless, high production costs and the tumorigenic risk associated with viral vectors will always be major hurdles. Reckhenrich *et al*. reported an attempt to avoid uncontrolled interactions of vectors with the *in vivo* environment by introducing copolymer-protected gene vectors into Integra®. The gene product stimulated the expression of VEGF in the dermal scaffolds and resulted in enhanced vascularization of full-thickness skin wounds in mice [47].

Additionally, VEGF-driven angiogenesis was achieved by Scherer *et al*. with a different approach. Poly-*N*-acetylglucosamine nanofibers were applied to scaffold-like membranes, which promoted wound healing in diabetic mice. The effect was correlated with increased numbers of blood vessels in the newly synthesized tissue [48].

##### Introducing an inducer for the proangiogenic factors

Some materials have the ability to induce the production of proangiogenic proteins. In an interesting study, the effect of copper-doped (Cu-doped) and undoped borate bioactive glass (BG) microfibers on angiogenesis was investigated. The ionic dissolution product of the Cu-doped borate BG microfibers stimulated *in vitro* EC migration and proliferation, formation of tube-like structures of human umbilical vein ECs (HUVECs), secretion of VEGF and upregulation of angiogenic-related genes in fibroblasts. In addition, Cu-doped BG microfibers were assessed in the treatment of full-thickness skin defects in a rodent model. They showed a better capacity than the undoped microfibers to improve maturation, deposition and arrangement of collagen fibers to resemble normal skin. This data suggested a positive effect of the Cu-doped microfibers on extracellular matrix remodeling and the healing of full-thickness skin wounds. The long-term effect and adverse reaction of Cu in this configuration has yet to be evaluated [49]. Another hybrid scaffold comprising a PLGA knitted mesh of collagen–chitosan was tested in a different study. Following implantation in rats, the scaffold augmented both the elastic strength of the newly formed skin and the density of its microvascular networks when compared with a scaffold comprised of collagen–chitosan alone [50]. Sun *et al*. studied physically modified dextran hydrogels and compared them with Integra® in full-thickness burn mouse models. The angiogenic response to cross-linked hydrogels was more robust compared to that of Integra® [51]. Although these studies provided evidence for the potential of structurally altered biodegradable hydrogels as scaffolds for skin regeneration in animal models, clinical studies are required to explore the clinical outcomes of the use of hydrogels for burns and other types of acute skin injuries. Furthermore, the controlled release of growth factor is crucial. Otherwise, there would be a risk of affecting the cell cycle, with uncontrolled tissue growth and potential tumor formation. Several strategies can be followed, besides calculation of the locally available dose, including degradation of the construct or the vehicle [52].

### Different types of cells involved in vasculogenesis

The cells involved in the construct represent a major factor in controlling angiogenesis, not only by their direct contribution to vessel formation but also by their secretion in the surrounding milieu. In a clinical study conducted by Hohlfeld and colleagues, cultured fetal skin fibroblasts embedded within sheets of insoluble collagen were tested in young children with burns. The outcomes included rapid healing with aesthetically satisfactory results, represented by the lack of scarring and total avoidance of autografting. The authors attributed the success of this approach to the biological activities of the growth factors transiently secreted by the donor fibroblasts in the wounds [53]. Various cell types were used in different systems to investigate neo-vessel formation, which made the comparison of their efficacy difficult. Furthermore, although the general direction of these studies is directed towards autologous cell use; some studies discussed the use of allogenic cells. The latter has shown a promising effect when injected in a burn model, in terms of healing and new vessel formation [54]. However, allogenic cells are less likely to reach the clinic due to several technical challenges, including production in enough numbers, standardization of production and characterization and unexpected immunological events [55].

#### Human dermal microvascular ECs

have been extensively studied and are considered safe for clinical use in skin tissue engineering, especially after the development of a serum-free culture method. These cells can be isolated from skin samples or neonatal foreskin with difficulty. The insufficient yield remains a challenge which requires up to 6 weeks for *in vitro* expansion. In addition, the cells are usually contaminated with fibroblasts, which hinder their use in clinical settings [56, 57].

#### The combination of fibroblasts and HUVECs

in a hyaluronic acid-based scaffold is currently used to study angiogenesis *in vitro* [58]. When the same mixture of cells is seeded onto a collagen/glycosaminoglycan matrix, a capillary-like meshwork develops. The density of this meshwork can be enriched by adding fibroblast growth factor or VEGF [59]. When HUVECs were combined with fibroblasts and keratinocytes in a collagen matrix, the constructed structure was able to develop a well-ordered vascular network. This outcome can be explained by the ability of fibroblasts to synthesize extracellular matrix proteins as a navigation grid for the migration of HUVECs as a countermeasure for the presence of collagen [60]. Upon transplantation in mice, pre-vascularized skin substitute was inosculated, resulting in functional hybrid vessels with *in situ* capillaries, 4 days after transplantation [61]. Both HUVECs and human microvascular ECs have exhibited similar revascularization capacities [62]. Subsequently, HUVECs have been considered a robust source of ECs with proven capability of capillary morphogenesis. However, these cells have two potentially critical limitations, which are their allogeneic origin and their limited proliferation potential, lacking the capacity to generate sufficient numbers of cells for human applications [63].

#### Unipotent endothelial progenitor cells

were identified by Asahara *et al*. 1997 as circulating progenitor cells for the endothelial lineage. These cells are of bone marrow origin, characterized by the upregulation of EC surface markers and downregulation of hematopoietic markers and contribute to *in vivo* adult vasculogenesis. Two major types of endothelial progenitor cells were identified regarding their functional characteristics and isolation kinetics: early outgrowth endothelial progenitor cells and late outgrowth endothelial progenitor cells [64].

##### Early outgrowth endothelial progenitors

originate from the monocytic cells (CD14+) and can be isolated from the mononuclear fraction of peripheral blood (PB) or umbilical cord blood (UCB), with higher yield from the latter. The colony forming unit ECs appear after 4–9 days of culture and can be characterized by their limited proliferation capacity. The early outgrowth endothelial progenitor cells contribute to the regulation of the angiogenic response through the production of growth factors without their incorporation into the endothelial intima [65]. Application of early outgrowth endothelial progenitor cells improves angiogenesis and epidermal wound healing, which makes these cells possible candidates for skin tissue engineering. Despite their defined angiogenic potential, ease of harvest and trophic effect on wound healing, their impaired proliferation capacities may hinder their clinical applicability [66].

##### Late outgrowth endothelial progenitors,

known also as blood outgrowth ECs (BOECs), exhibit augmented proliferation capacity than early outgrowth endothelial progenitors. Unlike early endothelial progenitors, BOECs can actively integrate into intimal endothelium and expand *in vitro* with relative ease [67, 68]. BOECs can contribute to vessel formation, as well as stimulate host angiogenesis indirectly through the secretion of a range of growth factors that modulate vasculogenesis and wound healing. Many studies have emphasized the potential of UCB-derived BOECs (UCB-BOECs), due to their great proliferative and vasculogenic potential [67]. However, the application of UCB-BOECs may not be risk-free for clinical settings as they are prone to karyotypic aberrations, despite the lack of tumorigenic evidence in the literature [69]. Moreover, the persistent risk of immune rejection with allogeneic UCB-BOECs will always be present unless autologous UCB-BOECs are made available through the use of cell-banking technology [70]. On the other hand, PB-BOECs have successfully been used in vascularization of a de-cellularized dermal skin construct, in which they remained traceable for more than 4 weeks *in vivo* [71], in the endothelialization of a de-cellularized aorta in sheep [72] and in blood vessel growth in tissue-engineered bone scaffolds [73]. Another study reported improved re-epithelialization, wound vascularization and dermal matrix organization of the wound area upon the application of PB-BOECs cultured onto a dermal fibroblast matrix. Interestingly, PB-BOECs were also found to induce coverage of the vessels with smooth muscle cells/pericytes [25]. Hence, PB-BOECs can revive the possibility for a fully autologous skin tissue engineering approach, since both, dermal fibroblasts and PB-BOECs can be extracted from an individual patient, expanded in culture and incorporated in a skin substitute. Equivalently the factors secreted by early outgrowth endothelial progenitors were found to stimulate the capacity of BOECs to form capillary tubes [74], which highlights the advantage of combining both cell types. This synergistic effect has been confirmed by revascularization in an ischemic limb model when early outgrowth endothelial progenitors were co-transplanted with BOECs [75].

#### Mesenchymal stem cells

(MSCs) are a heterogeneous group of multipotent progenitor cells that can be recovered from various tissues, including bone marrow, adipose tissue, skeletal muscles, umbilical cord (tissue and blood) and amniotic fluid, in addition to other fetal tissues [76]. Although they have a great differentiation potential, MSCs can have variability in their differentiation ability between subjects as well as across differentiation lineages. This challenge can—at least partially—be explained by the epigenetic signature obtained over the years [77]. The use of epigenetic modifiers as additives to differentiation protocols was associated with enhanced MSC differentiation into several targets [78–80]. To our knowledge, no similar studies have been conducted on epidermal cells yet. MSCs influenced the healing of deep burns in rats through various mechanisms, including differentiation to replace the damaged cells, decreasing inflammatory cell infiltration into the wound bed, accelerating neovasculogenesis and granulation tissue formation, maintenance of the extracellular matrix and the production of cytokines and growth factors [13]. Yoshikawa *et al*. reported improved vascularization when bone marrow-derived BM-MSCs were seeded in a collagen sponge [81]. Such an effect can be—at least partially—attributed to their trophic effect on host vessels [82]. Likewise, a synergistic influence on vascularization was reported *in vivo* upon combining bone marrow-derived hematopoietic cells with BM-MSCs [83]. Furthermore, BM-MSCs enhanced vascularization in a dermal scaffold in pig models [10] and accelerated burn and chronic wound healing when merged with artificially developed dermis in preliminary clinical trials [81]. A similar effect was reported on acute wounds when combined with a fibrin glue spray [84].

##### Adipose-derived MSCs

(Ad-MSCs) also have the ability to differentiate into ECs. Using various modifications of the original isolation protocols, a higher yield of MSCs can be isolated in a heterogeneous population of vascular cells that includes pericytes and endothelial progenitor cells. Ad-MSCs enhance the stability of the blood vessels while differentiating toward perivascular cells [85]. From a technical perspective, these cells are easy to harvest in abundance and can be applied in an autologous manner. Moreover, they exhibit considerable capacity for expansion *in vitro* [86]. Ad-MSCs were successfully implemented in skin tissue engineering, resulting in enhanced wound closure and improved vascularization [87]. Another study reported that Ad-MSCs stimulate fibroblast activity through the secretion of paracrine factors that upregulate the synthesis of matrix protein and improve the re-epithelialization *in vivo* [88].

##### USB-derived MSCs

(UCB-MSCs) can be differentiated *in vitro* to give rise to cells of all three germ layers, including cells with endothelial properties [89, 90]. However, when UCB-MSCs are applied to a hind limb ischemia model, the effect on myogenesis is clear but the effect on vascularization is controversial [91, 92].

#### Multipotent adult progenitor cells

(MAPC) cells are a subset of BM-MSCs that have distinctive molecular, morphological and functional characteristics. These cells showed potential to differentiate into both arterial and venous ECs *in vitro*. Nevertheless, the procedure required to harvest MAPCs is extremely lengthy, which, in turn renders autologous application of MAPCs difficult to achieve [93].

#### Induced pluripotent stem cells

(iPSCs) arise by delivering the potency genes, through transfection, to the patient’s somatic cells. Thus, iPSCs acquire the pluripotent characteristics of embryonic stem cells while avoiding any ethical ramifications or immunological reaction [94]. Analysis of several iPSC studies showed that CD31+, CD34+ and CD43− fractions were able to provide *in vitro* vascular models [95]. Prior studies have also shown that iPSC-ECs are capable of forming vessel-like structures both *in vitro* and *in vivo* within supporting matrigel matrices [96]. These studies brought more attention to iPSC-ECs as a clinically relevant cell source with satisfactory vascularization potential. iPSC-EC- or HUVEC-coated beads were co-embedded with normal human lung fibroblasts in a 3D fibrin matrix to assess their ability to form stable micro-vessels. Both HUVECs and iPSC-ECs formed vessel-like networks with some characteristics of mature microvasculature and utilized similar proteolytic invasion mechanisms. However, significant attenuation of sprouting and reduction in capillary network formation were reported with iPSC-ECs compared to HUVECs, as explained by differences in the expression levels of Matrix Metallopeptidase 9 [97]. Another study was conducted by Margariti *et al*. to generate a population of partial-iPSCs (PiPS) from human fibroblasts through short-term reprogramming over a 4-day transfection procedure with the four reprogramming factors (OCT4, SOX2, KLF4 and c-MYC) to human fibroblasts. The developed PiPS cells displayed the potential to differentiate into ECs in response to defined media and culture conditions and without the risk of forming tumors *in vivo*. PiPS-ECs showed improved neovascularization and promoted higher tissue blood flow recovery when injected intramuscularly into hind limb ischemic legs in a severe immunodeficient mouse model in comparison with control (no cells injection) and fibroblasts. PiPS-ECs also displayed significantly higher capillary numbers in comparison with fibroblasts when staining with CD31 antibodies. PiPS-ECs displayed enhanced engraftment ability and typical vascular architecture, whereas the fibroblasts control showed a random pattern and no vascular structures. Furthermore, PiPS-ECs displayed the ability to participate in tissue regeneration when they were used for the preparation of tissue-engineered vessels. When PiPS-ECs were seeded on de-cellularized vessel scaffolds prepared in a specially constructed bioreactor they exhibited robust attachment, stabilization and typical native-vessel architecture, with multiple layers of smooth muscle cells and an EC layer [98]. Several challenges still face iPSCs before clinical application, including chromosomal instability, expression of oncogenic genes and risk of teratoma formation. These risks are closely related to the production process of iPSCs with viral vectors [99].

## Conclusions

In skin tissue engineering, several strategies can be followed to promote vascularization. The use of biomaterials, the application of growth factors or the integration of a specific type of cells can enhance new capillary formation. Proper vascularization is essential for the uptake of any tissue construct, with special consideration of the skin, as the blood vessel access is only from underneath, while the rest of the organs can are vascularized from any surrounding surface. The greatest challenge is to achieve adequate vascularization, as the cells within the construct would typically have high metabolic demands during the initial stages of grafting. The vascular neoformation must eventually subside and should be limited to the physiological vascularization limit of normal skin. Hence, longer-term preclinical studies are required to evaluate the aspect of vascularization rate. In the meantime, successful integration of these vessels into a construct is a critical threshold for turning a conceptualized perfused construct *in vitro* to a fully vascularized tissue *in vivo*. This balance will be a future research necessity prior to the application of large-scale regenerative skin solutions.

## Data Availability

Not applicable.
